# When equal resources fail to produce equal opportunity: a critical narrative mini review of implementation equity in school-based physical activity for adolescents

**DOI:** 10.3389/fpubh.2026.1962860

**Published:** 2026-09-02

**Authors:** Jianfang Liu, Shuting Yin, Wei Wei, Weiwei Zhang

**Affiliations:** 1 Nanchang JiaoTong Institute, Nanchang, China; 2 Zhengdong New District Education, Culture, and Sports Bureau of Zhengzhou City, Zhengzhou, China; 3 Zhengzhou College of Finance and Economics, Zhengzhou, China; 4 Beijing College of Finance and Commerce, Beijing, China

**Keywords:** adolescents, health inequities, implementation equity, implementation science, intersectionality, low- and middle-income countries, narrative review, school-based physical activity

## Abstract

Schools are the most universal platform for promoting adolescent physical activity (PA), yet uniform mandates have not produced equal opportunities. This Mini Review is a critical narrative (integrative) review: its objective is conceptual integration across three literatures that have developed separately, participation disparities, implementation determinants, and PA policy analysis, rather than pooled effect estimation, and it is reported in line with SANRA. We searched PubMed and the Web of Science Core Collection for evidence published between January 2021 and June 2026, supplemented by seminal sources identified through citation tracking, to examine implementation equity: the fair distribution of organizational capacity, accountability, and support required to convert policy and resources into sustained, high-quality activity opportunities. Existing syntheses have separately quantified participation inequities, appraised policy–practice associations, and evaluated intervention scale-up; none has made the school’s organizational capacity to convert resources into opportunity the equity-relevant unit of analysis, and evidence from low- and middle-income countries (LMICs) remains peripheral to this literature. Three conclusions emerge. First, participation inequalities remain substantial and intersectional, with socioeconomic disadvantage, gender, disability, race and ethnicity, migration background, and rurality combining to restrict access. Second, implementation capacity is often distributed inversely to need. Schools serving disadvantaged populations face lower organizational readiness, staffing and time constraints, competing academic priorities, and policies that frequently lack funding and accountability, a pattern documented in high-income systems and LMICs alike. Third, implementation support, including school champions and teams, cross-sector partnerships, co-design with marginalized groups, and structured adaptation during scale-up, can strengthen delivery in high-need settings. However, equity effects remain poorly evaluated because studies emphasize reach and effectiveness while rarely assessing representative adoption or long-term maintenance. We identify four unresolved controversies and argue that equity should be treated as a primary implementation outcome rather than a background covariate. Equity-explicit reporting, intersectional analysis, participatory governance, proportionate support, and cross-sector accountability are needed to ensure that equal policy commitments generate genuinely equitable opportunities for adolescent physical activity.

## 1 Introduction

Adolescence is the decisive window for establishing lifelong movement habits, yet roughly four in five adolescents aged 11–17 worldwide fail to meet the World Health Organization (WHO) recommendation for daily moderate-to-vigorous physical activity (PA), a proportion scarcely improved in two decades (1, 2). Insufficient PA tracks into adulthood and contributes to cardiometabolic, musculoskeletal, and mental health burden (2, 3). The deficit is global but uneven: across 57 countries in the Global Matrix 4.0, overall PA scored the lowest average grade of ten indicators, varying with Human Development Index and region (4). Because schools reach almost every child within an already structured timetable, they are the pre-eminent setting for population-level PA promotion and are designated as such in global and national policy (5–7). Governments have responded with mandates: minimum physical education (PE) minutes, recess requirements and whole-of-school programmes (8, 9). A structural contradiction has nonetheless become visible. Uniform mandates do not yield uniform opportunity. Policy text and school-level practice diverge, and the divergence is socially patterned, being widest precisely in schools serving the most disadvantaged students (9, 10). Equal resources coexist with unequal opportunity.

That contradiction is global. Socioeconomic position, gender, disability, ethnicity and rurality pattern participation in high-income countries (11–14), and the same axes operate in low- and middle-income countries (LMICs), compounded there by weaker implementation infrastructure. Report cards from Mexico and Chile record overall PA grades of D or lower alongside marked sex differences (15, 16); few sub-Saharan African countries have a national PA plan covering school-age children (17); and PA interventions in China are overwhelmingly urban, excluding rural, ethnic-minority, left-behind and migrant children (18). Stakeholder studies in Nigeria, Thailand and Brazil identify one constraint set: absent funding, misaligned indicators, teacher workload, thin professional development as the operative barrier to delivery (19–21). Because expected gains from PA promotion are greatest in LMICs (22), the concentration of this research in high-income countries is itself an equity problem.

Public health theory anticipated this. Frohlich and Potvin argued that population-wide interventions can widen disparities because groups differ in their capacity to convert delivered resources into health (23); White and colleagues formalised the phenomenon as intervention-generated inequality (24, 25), and Hart’s inverse care law described the same inversion in medical care (26). What these accounts leave implicit is the organisational layer: in schools, the conversion of resource into opportunity is performed by an institution with finite readiness, staffing and attention, whose capacity itself is socially patterned. We term this implementation equity. The relevant evidence exists but is partitioned: meta-analysis has quantified participation inequities in organised sport and PA (11); reviews have catalogued access interventions (27), appraised policy–practice associations (8), and assessed reach, adoption and maintenance at scale (28); equity-lens reviews have mapped intervention coverage in China (18) and policy uptake in sub-Saharan Africa (17); and implementation science has begun to centre equity, though largely in healthcare (29–31). None treats the school’s capacity to convert provision into opportunity as the equity-relevant unit of analysis, so three questions fall between them: whether conversion capacity is itself socially patterned; whether equity is an implementation outcome or a subgroup covariate; and whether strategies that raise delivery on average do so in low-capacity schools. This Mini Review addresses that gap. It defines implementation equity and locates it between resource and participation equity; assembles the mechanisms of conversion failure and their gradient with school disadvantage into one account (Table 1); appraises implementation strategies by their equity properties rather than their average effect; and specifies what equity-explicit reporting requires.

**Table 1:** Equity in school-based physical activity: (A) Axes of participation disadvantage and the evidence establishing them; (B) Mechanisms of conversion failure in school-based physical activity, their equity gradient and candidate levers.

Axis of disadvantage	Representative evidence	Implication for provision
Panel A. Axes of participation disadvantage under nominally equal provision		
Socioeconomic position	Meta-analysis of 104 studies (163 effect sizes), high-income countries: children and adolescents from higher-socioeconomic-status households more likely to participate in organised sport (OR 1.87, 95% CI 1.38–2.36) and to meet PA guidelines (OR 1.21, 95% CI 1.09–1.33), the disparity larger for sport than for total PA (11, 12). Two rural US communities, children paying full price for school lunch had almost four times the odds of youth sport participation of those receiving free or reduced-price lunch (OR 3.91, 95% CI 1.95–7.80) (13).	Cost and organised-sport pathways ration access before school provision begins; school PA is the only channel that reaches both groups.
Sex and gender	Gap stable across cultures and widening with age (13, 48). Systematic review of systematic reviews: insufficient peer, family and teacher support and time scarcity the most frequently reported barriers for adolescent girls (49).	Provision must address the social environment of participation, not only its supply.
Disability and neurodevelopmental difference	US National Survey of Children’s Health 2021–2023: transition-age youth with autism spectrum disorder, intellectual disability or both had adjusted ORs for weekly PA of 0.32, 0.49 and 0.51, and for organised sport participation of 0.20, 0.31 and 0.24 (38). Aotearoa New Zealand stakeholder analysis: communication, transport, equipment cost, low awareness and inadequate social support the principal barriers; supportive school policy and inter-provider collaboration the principal facilitators (51).	Inclusion requires specified adaptation and inter-agency coordination, not general provision.
Migration background, ethnicity and racialisation	Girls with migration backgrounds in socioeconomically disadvantaged Swedish areas: gendered norms, economic constraint and cultural expectation compound (52). St. Louis region: lower girls’ sport diversity where the proportion of African American residents was higher (53). Canada 2022 report card: substantial data gaps for disabled, Indigenous, 2SLGBTQ+, newcomer and racialised young people (14).	Groups least visible in surveillance are frequently the least served; disaggregated reporting is a precondition of equitable provision.
Intersecting identities	62,940 US adolescents: lowest participation among those holding several marginalised identities simultaneously (low resource access, questioning gender identity, sexual-minority status, non-White ethnicity); lowest subgroup 12.8% (54).	Single-axis targeting systematically misses those furthest from provision (55, 56).
Geography and national context	Mexican and Chilean report cards: overall PA graded D or lower alongside marked sex differences (15, 16). China: of 65 PA interventions, almost all were urban and only two rural, excluding low-education families, ethnic minorities and left-behind and migrant children (18). Sub-Saharan Africa: few countries have a national PA plan for school-age children (17).	Neighbourhood conditions and national implementation infrastructure set the ceiling on what schools can deliver (57).

Mechanism	Representative evidence	Gradient with school disadvantage	Candidate lever
Panel B. Mechanisms of conversion failure at school level			
Organisational readiness	Motivation, innovation-specific and general capacity modelled as multiplicative; relationships, competing priorities, visible benefit and staff buy-in identified as school-level determinants (42, 43)	Readiness sub-components are depressed simultaneously in high-need schools; turnover fractures the relationships that implementation depends on	Assess readiness before rollout; establish implementation teams and wellness champions (69, 70)
Competing academic priorities	PE time displaced and recess shortened for examination preparation (19, 60, 61); misaligned performance indicators and inadequate budget reported by Thai stakeholders (20)	Attainment pressure is greatest in schools serving the lowest-attaining intakes, so PA is displaced first where need is highest	Align school performance indicators with PA requirements; protect timetabled minutes in law
Workforce capacity	Role-breadth self-efficacy shaped by occupational and organisational socialisation (62); teaching workforce among the only significant positive predictors of implementation level (63)	Staffing shortfalls, turnover and limited professional development concentrate in under-resourced schools	Pre-service and in-service development for CSPAP coordination roles (47, 62)
Time, facilities and financing	Time scarcity the most frequently reported barrier in US and Swedish stakeholder studies (61, 64); teacher workload the leading difficulty in Brazil (21)	Ageing facilities and thin community assets cluster with school disadvantage, compounding staffing deficits	Paid teacher time; shared community facility agreements; dedicated programme financing
Policy accountability and funding	Only 25.5% of US state PE and 22.7% of recess laws contain accountability provisions and almost none contain funding (9); Quebec mandates equalised availability across deprivation levels (65)	Without monitoring or money, compliance tracks pre-existing capacity, reproducing the inverse care law organisationally	Attach accountability provisions, financing and technical assistance to every mandate (9, 65)

PA, physical activity; PE, physical education; CSPAP, Comprehensive School Physical Activity Program.

## 2 Review type and evidence base

This article is a critical narrative (integrative) review, prepared under the Frontiers Mini Review article type. Its objective is conceptual integration, defining implementation equity and joining three literatures that use incompatible designs and outcome definitions, rather than pooled effect estimation, for which a systematic review would be the indicated design (32, 33). Selection is therefore purposive and the synthesis interpretive; this is not a systematic review and should not be read as one. Reporting follows SANRA, the instrument developed for narrative reviews (34); PRISMA and PRISMA-ScR are the reporting guidelines for systematic and scoping reviews, respectively (35, 36) and do not apply to this article type.

We searched PubMed and the Web of Science Core Collection for records published between 1 January 2021 and 30 June 2026, combining four concept blocks: population; setting (adolescents, children, school); exposure (physical activity, physical education, sport, recess, active transport); and analytic focus (equity, disparity, implementation, policy, scale-up), limited to peer-reviewed English-language articles; the strings as executed are given in Supplementary Tables S1 and S2. Records were eligible if they concerned school-age children or adolescents, addressed school-based PA, PE, recess, school sport or active transport, and reported on equity or disparity, implementation determinants or outcomes, PA policy, or scale-up; the full criteria are given in Supplementary Table S3. Titles and abstracts were screened against these criteria and potentially eligible full texts assessed. Because Mini Reviews are selective by design rather than exhaustive, we prioritised systematic reviews and meta-analyses, national surveillance and report-card studies, policy analyses, and implementation evaluations using established determinant or evaluation frameworks, with deliberate inclusion of LMIC evidence. Seminal works predating 2021 (foundational statements on health equity, implementation determinant frameworks, and theory on intervention-generated inequality) were identified by citation tracking and are cited without date restriction to provide theoretical grounding (Supplementary Table S4).

## 3 From equal resources to equal opportunity: defining implementation equity

Equality denotes identical inputs; equity denotes fair outcomes given differing starting points, needs and constraints (27, 37, 38). Uniform PE minutes, standard facility specifications and identical programme rollouts satisfy equality; they satisfy equity only if every school can convert them at the same rate.

We define implementation equity as the fair distribution, across schools and student subgroups, of (a) the organisational capacity to translate policy and resources into delivered, high-quality opportunity and (b) the accountability and support structures sustaining it. It sits downstream of resource equity and upstream of participation equity. The diagnostic question is not whether a resource was provided, but whether an opportunity was realised: for whom, at what quality, and for how long.

Three consequences follow. First, the implementation gap is a systematic mediator, not random noise. Across US states, 96.1% had PE laws and 43.1% recess laws, yet only 25.5% of PE and 22.7% of recess laws contained accountability provisions, and almost none contained funding (9). Policy without accountability or funding is largely symbolic, and symbolic policy is converted only by schools already able to convert.

Second, established frameworks become equity diagnostics once their outputs are disaggregated: the reach and adoption dimensions of RE-AIM ask who was reached and which settings adopted (28, 39); CFIR locates determinants across inner and outer setting (40, 41); R = MC2 models readiness as motivation × innovation-specific × general capacity (42, 43); the Interactive Systems Framework separates delivery, support and synthesis systems (44, 45); and the Health Equity Implementation Framework makes this layer explicit (29, 31, 46).

Third, delivery models differ in their equity properties. Whole-of-school approaches and Comprehensive School Physical Activity Programs (CSPAP) distribute activity across PE, recess, classroom time, before- and after-school programmes and active transport (10, 47). Because they demand more coordination, they are more sensitive to capacity, and can widen gaps: across 162 US elementary schools, whole-of-school PA index scores fell as the proportion of economically disadvantaged students rose (10). The most demanding model is weakest where need is greatest.

## 4 Who remains absent under nominally equal provision

Participation gradients are large, replicable and intersecting. The principal axes, the evidence establishing each, and what each implies for provision are set out in Table 1, Panel A; three analytic points follow.

First, the gradients are steep and consistent. Socioeconomic position is among the strongest single predictors, in high-income countries (11–13) and in LMICs alike (15, 16, 18); the sex gap is stable across cultures and widens with age (13, 48, 49); disability and neurodevelopmental difference reduce the odds of both PA and organised sport participation by a factor of two to five (50, 51); and migration background, ethnicity and rurality each add a further layer (14, 52, 53). Second, these axes do not act independently. Intersectional analysis of 62,940 US adolescents found the lowest participation among those holding several marginalised identities simultaneously, the lowest subgroup reaching 12.8% (54); because marginalisations interact qualitatively rather than additively (55, 56), single-axis targeting will systematically miss those furthest from provision. Third, the least visible groups are often the least served: Canada’s 2022 report card documented substantial data gaps for disabled, Indigenous, 2SLGBTQ+, newcomer and racialised young people (14), and neighbourhood conditions add a geographic layer that school-level data rarely capture (15, 57).

Every study and statistic previously reported in this section is retained, in full, in Table 1, Panel A.

## 5 Why equal resources fail: mechanisms of conversion failure

Four school-level mechanisms explain why nominally equal provision yields unequal opportunity, and each is patterned by school disadvantage (Table 1, Panel B).

Organisational readiness is the proximal constraint. Applying the R = MC2 heuristic, qualitative work identified relationships, competing school priorities, visible benefit and staff buy-in as determinants of implementation (43). In high-need schools these sit low simultaneously: turnover fractures relationships, attainment pressure compresses time, benefits are less visible, and added workload meets resistance (43, 58). Implementation quality, not programme content, then becomes the binding constraint on outcomes (59).

Competing priorities operate everywhere but bite hardest where attainment pressure is greatest. PE time is displaced and recess shortened in favour of examination preparation (19, 60, 61). Stakeholders in Thailand and Nigeria identify misaligned performance indicators, low programme priority and absent budget as the principal barriers (19, 20). In both middle-income systems, the constraint is organisational rather than informational: the policy is known and cannot be delivered.

Workforce capacity determines delivery quality. PE teachers are the operational core of school PA, and their role-breadth self-efficacy is shaped by occupational and organisational socialisation; weak pre-service preparation, limited professional development and thin administrative support erode confidence in the coordinating roles CSPAP requires (62). Teacher workload in Brazil (21) and teaching-workforce capacity in China (63) emerge as the dominant predictors of implementation level. Time, facilities and funding form the material floor (61, 64).

Accountability and financing bind the others together. Where laws specify minimums but attach neither monitoring nor money, compliance depends on pre-existing capacity (9). The converse is instructive: in Quebec, mandated interventions were available in most schools regardless of deprivation level, whereas non-mandated interventions varied markedly (65). Mandates with teeth compress between-school variance; mandates without them do not. This is Hart’s inversion reproduced at the organisational level (23, 26), consistent with the fundamental-cause account in which flexible resources are redeployed to capture each new health-promoting opportunity (66).

## 6 What has been tried: strategies and their equity evidence

Whole-of-school multicomponent intervention is best evidenced, but its average effect is modest. A systematic review and meta-analysis of 61 secondary-school interventions found a statistically significant pooled effect only for body mass index (SMD -0.09), with no significant change in PA, sedentary time or waist circumference; only 51% of studies reported implementation-related factors (67). A RE-AIM-structured review of 43 school PA interventions found a moderate effect on cardiorespiratory fitness (SMD 0.28) but none on body fat, with effectiveness reported in 72.7% and reach in 53.1% of studies against maintenance in 4.7% (68). Weak average effects are hard to interpret when implementation is unmeasured.

Implementation support is the most promising lever. Across US schools, 60% had a wellness champion and 37% an implementation team, the presence of a team being associated with better school health climate (69). In a Philadelphia sports-based health intervention for underserved middle-school students, limited staff training, turnover, and rotating programme design were the principal barriers (58). In the Physical Activity 4 Everyone scale-up across 49 low-socioeconomic-status secondary schools, 69.6% of schools receiving implementation support delivered at least four PA practices at 24 months versus 0% of control schools (70). Structured implementation support can therefore raise delivery in exactly the schools where conversion normally fails.

Cross-sector partnership and co-design extend this. Teachers supported by community nutrition-education partners reported higher delivery frequency and fewer barriers (71), and the iPLAY scale-up reached roughly 31,000 students across 115 schools with small positive effects at 24 months (72). Co-design with the intended group, adolescent girls in a disadvantaged Irish community using the Behaviour Change Wheel (73, 74), girls from disadvantaged Swedish areas taking leadership roles (52), and Indigenous youth mentorship built on community-academic partnership (75) repositions participants from beneficiaries to co-designers.

Scale-up requires disciplined adaptation rather than replication. In PA4E1, 20 adaptations and 20 modifications occurred, most fidelity-consistent and judged to preserve effectiveness, driven mainly by available resources (76), while Resistance Training for Teens reached about 10,000 students across 249 schools with substantial contextual variation (77). Rapid-cycle adaptation during COVID-19 extended a classroom intervention statewide (78), while showing that digital delivery can itself be inequitably accessible. Almost all of this evidence originates in high-income systems, so the transferability of these strategies to LMIC schools, where the capacity deficits they address are typically larger, remains untested (17, 18).

## 7 Controversies and competing perspectives

Four disagreements remain unresolved.

Universal versus targeted provision. Universalists hold that common standards guarantee a floor, and the Quebec evidence supports them (65). Critics counter that identical standards applied to unequal capacity widen gaps (10, 23), and that proportionate universalism, universal provision with intensity scaled to need, is the resolution rather than a compromise.

Do mandates work? States with weekly PE-minute policies record substantially more PE time than states without (79), yet a systematic review found associations between school PA policies and actual practices or behaviour mostly non-significant or uncertain (8). The likeliest reconciliation is that mandate efficacy is conditional on accountability and financing, which most laws lack (9).

Individual versus structural attribution. Behavioural framings emphasise choice, education and skills; structural accounts emphasise that choice is constrained by income, safety, facilities and time (3). Longitudinal evidence from the China Health and Nutrition Survey suggests that returns to PA participation themselves differ by social class (80), a single-cohort finding that requires replication but that, if robust, would imply that behavioural intervention alone cannot close structural gaps.

Rights versus instrumental framing. Critical discourse analysis of global policy documents from 2004 to 2025 found UNESCO positioning PA as a human right while UN and WHO texts adopt an instrumental logic tied to development goals; children, adolescents and women receive prominent attention while migrants, refugees, ethnic minorities and older people are folded into a generic vulnerability category and LGBTQ+ populations almost disappear (81). Framing shapes who becomes visible, and therefore who is funded.

Underlying all four is a dispute about reporting standards. If studies report effectiveness under favourable conditions but not implementation in disadvantaged settings, the public health value of an intervention is systematically overestimated (28, 39, 68). Equity-explicit reporting, using frameworks such as PROGRESS-Plus (82), is a precondition for adjudicating the substantive debates.

## 8 Discussion

Equal distribution of resources is necessary but far from sufficient. Between policy text and student experience lies a process of organisational conversion whose uneven distribution is the proximal source of unequal opportunity. Because the schools least able to convert are those serving students with the greatest need, ostensibly neutral provision reproduces the gradient it was intended to close.

Four gaps constrain progress. Data are missing non-randomly: in the longest-running US fitness surveillance system, 20.1% of students lacked all three fitness measures between 2006 and 2020, rising to 36.3% in high school and concentrated among non-Hispanic Black students and the lowest-socioeconomic-status neighbourhoods (83); policy calibrated on such data is calibrated on the already-visible. Representation is skewed: of 65 PA interventions in China, almost all were urban and only two rural, excluding low-education families, ethnic minorities and left-behind and migrant children (18). Implementation reporting is thin, particularly for adoption representativeness and maintenance (28, 68). And equity is rarely designed in, remaining largely rhetorical in policy texts, unaccompanied by implementation tools, financing, accountability or participatory governance (18, 81).

Five priorities follow. First, treat equity as a primary implementation outcome rather than a background covariate, specifying equity objectives at design and disaggregating delivery and effect by subgroup (29, 30, 82). Second, adopt intersectional analysis, combining quantitative subgroup methods with qualitative work (52, 54, 56). Third, build participatory governance with young people, families and communities as co-designers (73, 75). Fourth, pursue structural reform beyond the school gate, linking education, urban planning, transport, health and sport, since schools cannot resolve determinants they do not control (57, 84). Fifth, generate implementation evidence at scale using hybrid effectiveness-implementation designs (85, 86), tested implementation strategies (87) and full external-validity reporting (39), and in LMIC as well as high-income systems (22).

Policymakers should abandon the assumption that legislation completes the task, and attach accountability provisions, dedicated financing and technical assistance to every mandate (9, 65). School leaders should treat PA as core educational mission rather than optional enrichment, investing in implementation teams, partnerships and health climate (43, 69). Researchers should report who was reached, who was missed, and what was sustained.

Inequity in PA opportunity shapes not only current health but, through behavioural trajectories and accumulated physical literacy, lifelong health stratification (3, 66). Converting equal distribution into equal opportunity requires a shift from fragmented programmes to equity-oriented systems, and treating adolescents as agents of that change rather than its targets.
