# Supplementary material for: When equal resources fail to produce equal opportunity: a critical narrative mini review of implementation equity in school-based physical activity for adolescents
Source: Front Public Health. 2026 Sep 2;14:1962860. doi: 10.3389/fpubh.2026.1962860 (PMC13579453; doi:10.3389/fpubh.2026.1962860)
Supplement: Supplementary file 1 [file Supplementary_File_1.docx]

**Supplementary Material**

**1. Overview**

This Mini Review used a structured, reproducible literature search combined with targeted citation tracking. Two components are distinguished throughout. The primary search covered contemporary empirical and policy evidence published within a fixed five-and-a-half-year window (1 January 2021 to 30 June 2026), and supplies all substantive empirical claims in the article. A secondary, undated component identified seminal conceptual and methodological works that provide the theoretical scaffolding for the argument; these were located by backward and forward citation tracking from records retrieved in the primary search, and are listed in Supplementary Table S4.

Because the Mini Review article type is explicitly selective rather than exhaustive, the search was not conducted as a systematic review and no formal risk-of-bias assessment or meta-analysis was performed. The procedure is reported here so that readers can judge the basis of selection and reproduce the retrieval step.

**2. Databases and search window**

Two databases were searched: PubMed (US National Library of Medicine) and the Web of Science Core Collection (Clarivate; Science Citation Index Expanded, Social Sciences Citation Index, and Emerging Sources Citation Index). PubMed was selected for coverage of biomedical, epidemiological and public health literature; the Web of Science Core Collection was selected for its coverage of education, sport science, sociology and policy journals that PubMed indexes incompletely, and for its cited-reference searching, which supported the citation-tracking component.

The publication window was 1 January 2021 to 30 June 2026 inclusive. The start date was chosen so that the review captures evidence published after the World Health Organization 2020 physical activity guidelines and after the first wave of COVID-19 school closures, both of which reset the policy and evidence context for school-based physical activity.

**3. Search strategy**

The search combined four concept blocks with the Boolean operator AND: (i) population, (ii) setting, (iii) exposure, and (iv) analytic focus. Within blocks, terms were combined with OR. Block (iv) deliberately merges equity terms with implementation and policy terms, because the review’s premise is that these two literatures address the same phenomenon from different directions and are rarely retrieved together.

**Supplementary Table S1.** PubMed search string, executable as written.

| **#** | **Concept block** | **Search string** |
| --- | --- | --- |
| 1 | Population | "adolescent"[MeSH] OR adolescen*[tiab] OR youth[tiab] OR  teenager*[tiab] OR "young people"[tiab] OR child*[tiab] OR  student*[tiab] OR pupil*[tiab] |
| 2 | Setting | "schools"[MeSH] OR school*[tiab] OR "physical education and  training"[MeSH] OR classroom*[tiab] OR recess[tiab] OR  playtime[tiab] OR "after-school"[tiab] |
| 3 | Exposure | "exercise"[MeSH] OR "motor activity"[MeSH] OR "sedentary  behavior"[MeSH] OR "physical activity"[tiab] OR "physical  education"[tiab] OR sport*[tiab] OR "active transport*"[tiab]  OR "active travel"[tiab] OR "moderate-to-vigorous"[tiab] |
| 4 | Analytic focus | "health equity"[MeSH] OR "healthcare disparities"[MeSH] OR  "socioeconomic factors"[MeSH] OR "health policy"[MeSH] OR  "implementation science"[MeSH] OR equit*[tiab] OR  inequit*[tiab] OR inequal*[tiab] OR disparit*[tiab] OR  "social gradient"[tiab] OR disadvantag*[tiab] OR  marginali*[tiab] OR implement*[tiab] OR "scale-up"[tiab] OR  "scaling up"[tiab] OR fidelity[tiab] OR adoption[tiab] OR  polic*[tiab] OR mandate*[tiab] OR legislat*[tiab] |
| 5 | Combination and limits | (#1 AND #2 AND #3 AND #4)  AND 2021/01/01:2026/06/30[Date - Publication]  AND english[Filter] |

**Supplementary Table S2.** Web of Science Core Collection search string, executable as written.

| **#** | **Concept block** | **Search string** |
| --- | --- | --- |
| 1 | Population | TS=(adolescen* OR youth OR teenager* OR "young people" OR  child* OR student* OR pupil*) |
| 2 | Setting | TS=(school* OR "physical education" OR classroom* OR recess  OR playtime OR "after-school") |
| 3 | Exposure | TS=("physical activity" OR "physical education" OR sport* OR  exercise OR "active transport*" OR "active travel" OR  "sedentary behavior" OR "sedentary behaviour" OR  "moderate-to-vigorous") |
| 4 | Analytic focus | TS=(equit* OR inequit* OR inequal* OR disparit* OR "social  gradient" OR disadvantag* OR marginali* OR implement* OR  "scale-up" OR "scaling up" OR fidelity OR adoption OR polic*  OR mandate* OR legislat*) |
| 5 | Combination and limits | #1 AND #2 AND #3 AND #4  Refined by: Publication Date 2021-01-01 to 2026-06-30;  Language = English; Document Types = Article OR Review |

**4. Eligibility criteria**

**Supplementary Table S3.** Inclusion and exclusion criteria.

| **Domain** | **Included** | **Excluded** |
| --- | --- | --- |
| Population | Children and adolescents of school age (approximately 5–19 years); or the schools, teachers and administrators serving them | Adults only; pre-school populations only; elite or performance-sport populations |
| Setting | Primary and secondary schools; school-linked provision including recess, classroom activity breaks, before- and after-school programmes, and active transport to school | Clinical or rehabilitation settings; community or club provision with no school linkage and no bearing on school-level equity or implementation |
| Exposure | Physical activity, physical education, organised sport, recess and sedentary behaviour in or around the school day | Nutrition, sleep or substance-use interventions with no physical activity component |
| Analytic focus (at least one required) | (i) equity, disparity or differential participation and opportunity; (ii) policy content, adoption, enforcement or accountability; (iii) implementation determinants, strategies, fidelity, adaptation, scale-up or sustainment | Efficacy or physiological studies reporting neither an equity dimension nor implementation data |
| Design | Systematic and scoping reviews, meta-analyses, randomised and cluster-randomised trials, quasi-experimental and observational studies, qualitative and mixed-methods studies, policy and document analyses, national surveillance and report cards | Conference abstracts; editorials and commentaries without substantive synthesis; study protocols (retained only where cited to illustrate trial design) |
| Publication | Peer-reviewed and published in English between 1 January 2021 and 30 June 2026; supranational policy documents (WHO, UNESCO, UN) and national report cards cited as primary policy sources | Non-peer-reviewed grey literature other than the policy sources specified; publications outside the window, except seminal works handled under Section 6 |

**5. Screening, selection and synthesis**

Records retrieved from both databases were exported to a reference manager and de-duplicated. Titles and abstracts were screened against the criteria in Supplementary Table S3, and the full text of every potentially eligible record was assessed. Consistent with the Mini Review article type, selection among eligible records prioritised, in descending order: (i) systematic reviews and meta-analyses; (ii) national surveillance systems and report cards; (iii) policy content and enforcement analyses; (iv) implementation evaluations using a named determinant or evaluation framework; and (v) primary studies providing geographic or population coverage not otherwise represented. Where several eligible records reported the same finding, the most recent and most methodologically comprehensive was cited.

**6. Identification of seminal literature**

The argument of this Mini Review is that a recognised problem in public health theory — the differential capacity of social groups to convert delivered resources into health — has an unexamined organisational analogue in schools. Making that argument requires conceptual sources that predate the search window. These were identified in two steps. First, the reference lists of all included reviews, framework applications and policy analyses were examined for repeatedly cited theoretical and methodological works (backward citation tracking). Second, the Web of Science cited-reference function was used to confirm that each candidate work remained actively cited in the contemporary school physical activity and implementation literature (forward citation tracking). Works meeting both conditions, and directly load-bearing for a specific claim in the article, were retained. Thirty such sources were included; they are listed in Supplementary Table S4 with the reason for inclusion.

**Supplementary Table S4.** Seminal sources identified outside the search window, with the function each performs in the argument. Reference numbers correspond to the reference list of the main article.

| **Ref. no.** | **Source** | **Function in the argument** |
| --- | --- | --- |
| 1 | Guthold et al. (2020) | Global prevalence of insufficient physical activity in adolescents; establishes the scale of the problem. |
| 2 | Bull et al. (2020) | WHO 2020 guidelines; defines the recommendation against which sufficiency is judged. |
| 4 | WHO (2018) | Global Action Plan on Physical Activity; designates schools as a priority setting. |
| 5 | Institute of Medicine (2013) | Foundational statement of the school’s role in physical activity provision. |
| 6 | Sallis et al. (2012) | Positions physical education as a public health instrument, not a curricular subject alone. |
| 10 | Fröhlich and Potvin (2008) | Core theoretical anchor: population approaches can widen disparities because groups differ in their capacity to convert delivered resources into health. |
| 11 | White et al. (2009) | Formalises intervention-generated inequality. |
| 12 | Lorenc et al. (2013) | Empirical evidence on which intervention types generate inequality. |
| 13 | Hart (1971) | The inverse care law; template for the inverse implementation pattern described in Section 5. |
| 14 | Baumann and Cabassa (2020) | The equity lens in implementation science; direct conceptual precursor to implementation equity. |
| 15 | Brownson et al. (2021) | Agenda-setting argument that implementation science must prioritise health equity. |
| 16 | Woodward et al. (2019) | Health Equity Implementation Framework; makes the equity layer of determinants explicit. |
| 17 | Whitehead (1992) | Original conceptual separation of equality from equity in health. |
| 18 | Braveman and Gruskin (2003) | Operational definition of health equity used in the article. |
| 20 | Glasgow et al. (1999) | RE-AIM; its reach and adoption dimensions are repurposed here as equity diagnostics. |
| 22, 23 | Damschroder et al. (2009, 2022) | CFIR and its update; the determinant framework used across the included implementation evaluations. |
| 24 | Scaccia et al. (2015) | R = MC² readiness heuristic, applied in the school studies reviewed. |
| 26 | Wandersman et al. (2008) | Interactive Systems Framework; separates delivery, support and synthesis systems. |
| 28 | Proctor et al. (2011) | Implementation outcomes taxonomy; the object of the equity-lens reframing. |
| 29 | Carson et al. (2014) | Conceptual framework for CSPAP; defines the delivery model whose equity properties are analysed. |
| 43 | Crenshaw (1991) | Intersectionality; grounds the claim that marginalisations interact qualitatively, not additively. |
| 44 | Bauer (2014) | Quantitative intersectionality methods for population health; supports the analytic recommendation in Section 8. |
| 47 | Durlak and DuPre (2008) | Establishes that implementation quality, not programme content, binds outcomes. |
| 57 | Phelan et al. (2010) | Fundamental cause theory; explains why flexible resources are redeployed to capture each new health-promoting opportunity. |
| 65 | Michie et al. (2011) | Behaviour Change Wheel; the framework used by the co-design study reviewed in Section 6. |
| 73 | O’Neill et al. (2014) | PROGRESS-Plus; the equity-reporting framework recommended for implementation studies. |
| 77 | Sallis et al. (2006) | Ecological model of active living; grounds the cross-sector recommendation. |
| 78 | Curran et al. (2012) | Hybrid effectiveness–implementation designs; underpins the fifth research priority. |
| 80 | Powell et al. (2015) | ERIC compilation of implementation strategies; the menu from which support strategies are drawn. |

The table lists 29 rows because Damschroder et al. is a paired citation (2009 and 2022), giving 30 seminal references in total.

**7. Limitations of the search**

Four limitations should be borne in mind. First, restriction to two databases and to English-language publication will have under-represented evidence from low- and middle-income settings and from national-language education and sport-policy literatures; this compounds the representation gap the article itself identifies. Second, the selective citation procedure appropriate to a Mini Review means that the cited evidence is illustrative rather than exhaustive, and no claim is made that the retrieved set is complete. Third, no formal risk-of-bias or certainty-of-evidence appraisal was performed, so the reported effect estimates are reproduced as their authors published them and should be read with the limitations of their source designs. Fourth, the seminal-source component is by construction a purposive selection; other theoretically defensible groundings for the same argument exist.
